# Can internet usage reduce health inequality among rural residents? Evidence from China

**DOI:** 10.3389/fpubh.2025.1743477

**Published:** 2026-01-09

**Authors:** Yanlin Peng, Jingjing Deng

**Affiliations:** 1 School of Economics, Hunan Agricultural University, Changsha, China; 2 College of Business, Hunan First Normal University, Changsha, China

**Keywords:** accessibility of healthcare services, affordability of healthcare services, health inequality, internet usage, rural residents

## Abstract

The widespread adoption of the internet has made its role in reducing health inequalities within the digital health domain increasingly clear. Using data from six waves of the China Family Panel Studies (CFPS) spanning 2012–2022, this study employs a two-way fixed effects model to systematically examine the impact of internet usage on health inequalities among Chinese farmers. The findings reveal three key insights: (1) the empirical results indicate that internet usage contributes to both improved health outcomes and greater health equity among farmers. Specifically, internet usage not only enhances farmers’ overall health status but also reduces health disparities. (2) Mechanism analysis demonstrates that the health-equity effect of internet usage operates through two primary pathways: narrowing health gaps by improving access to healthcare services and reducing health inequalities by increasing the affordability of these services. (3) Heterogeneity analysis reveals significant group-specific variations in the effect of internet usage on health inequality. Notably, the mitigating effect is more pronounced among young adults, those with moderate educational levels, and those with low healthcare expenditures, while its influence is relatively limited in other farmer groups. This study provides robust evidence that internet usage can reduce health inequalities among farmers and offers important insights for developing targeted policies to reduce health disparities.

## 1 Introduction

Health is the foundation of socioeconomic development worldwide. A robust national health system significantly enhances labor productivity, fostering long-term economic prosperity. In China’s distinctive urban–rural dual structure, rural health concerns extend beyond public health and represent a major national challenge of equity and sustainable development. As the backbone of rural revitalization, the health and well-being of farmers not only determine the quality of life and family happiness for hundreds of millions but also profoundly influence economic growth and social stability across vast rural regions. Healthy farmers are the core workforce for agricultural production and rural industry, with their health capital serving as a fundamental source of vitality for rural economic growth. Therefore, improving farmers’ health has long been a strategic priority for the Chinese government.

While improving farmers’ health has long been a government priority, the persistent urban–rural gap in medical resource allocation continues to contribute to significant health inequality among rural residents. According to the China Statistical Yearbook, by the end of 2023, rural areas had 71 healthcare technicians, 27 licensed (assistant) physicians, 31 registered nurses, and 65.23 hospital beds per 10,000 people. These numbers are considerably lower than those in urban areas, which stood at 109, 41, 51, and 80.17, respectively. Many rural areas also face additional challenges, including shortages of high-quality medical resources, limited access to healthcare, inconsistent service quality, and low health awareness among residents. In this context, the rapid expansion of internet infrastructure offers a potential pathway to bridge this gap. According to the National Bureau of Statistics of China, by the end of 2024, more than 90% of administrative villages had achieved 5G network coverage, rural internet penetration had reached 67.4%, and the number of rural internet users continued to grow. Online healthcare platforms now facilitate remote professional consultations and diagnoses, while various health management applications enable farmers to monitor their health in real time and implement lifestyle interventions. These developments suggest that internet use may serve as a promising tool to improve farmers’ health and reduce health inequality.

However, the potential of the internet to improve health outcomes is not without challenges. The digital divide remains a significant barrier among the rural population, with notable differences in internet access, usage patterns, and the distribution of benefits (1). Factors such as age, income, education level, digital literacy, and regional infrastructure have created substantial gaps in both access and skills (2). While some farmers are able to effectively utilize internet tools to obtain health information and services (3), others remain largely excluded from these digital health resources (4, 5). This disparity raises important questions that require empirical investigation: Does internet use actually improve the overall health of farmers? Does it function as an inclusive health equalizer that narrows existing disparities, or does it create a new health divide? Could the uneven distribution of digital capabilities reinforce or even exacerbate health inequality within the rural population? Addressing these questions is essential for understanding the true impact of internet use on rural health and for informing the design of effective digital rural policies and health promotion strategies (6).

## 2 Literature review

The relationship between internet use and health inequality has attracted considerable scholarly attention. Existing research presents two contrasting perspectives: one emphasizes the role of the digital divide in exacerbating health disparities, while another highlights the compensatory potential of internet use in reducing such disparities.

### 2.1 The exacerbating effect of internet usage on health inequality

One strand of literature suggests that internet use may widen health inequalities through three interrelated mechanisms: the access gap, the usage gap, and the benefit gap.

The access gap refers to structural disparities in internet connectivity and device ownership that directly limit access to online health resources for disadvantaged populations. Research has documented that racial and ethnic minorities face restricted access to health information due to differences in device dependence (7), and that low-income adolescents experience constrained health information channels as a result of inadequate internet access (8). In the Chinese context, Long et al. found that urban–rural disparities in digital infrastructure resulted in substantially lower mental health benefits from internet use for rural residents compared to their urban counterparts (9). Similarly, studies in European settings have identified a compounded disadvantage among low-income groups who face barriers to both health resources and digital access (10).

The usage gap concerns differences in internet skills and usage patterns across socioeconomic groups. While individuals with higher socioeconomic status tend to use the internet for instrumental purposes such as health management, those with lower status engage primarily in entertainment activities, thereby widening the health literacy gap (11, 12). Neter and Brainin introduced the concept of the e-health literacy gap, demonstrating that individuals with limited literacy face greater risks of misinterpreting health information (13). Additional research has shown that underrepresentation in online health communities further limits access to social support for marginalized groups (14, 15).

The benefit gap reflects the uneven distribution of health gains from internet use, even when access and skills are present. For example, Niccodemi et al. found age-based disparities in mental health outcomes, with internet use worsening depression among adults aged 65 to 70 but alleviating symptoms among those over 80 (16). Studies have also demonstrated that individuals with severe mental illness experienced heightened negative emotions during internet use, suggesting that digital platforms may intensify health disadvantages for vulnerable populations (17–19). Collectively, these three dimensions of the digital divide suggest that internet use can exacerbate existing health inequalities.

### 2.2 The equalizing effect of internet usage on health inequality

An alternative perspective holds that internet use can address structural deficiencies in traditional healthcare systems through three compensatory channels: information, services, and support.

Regarding information compensation, internet use has been shown to disrupt traditional monopolies on health information and reduce information asymmetry for disadvantaged groups (20). Tian, drawing on the digital dividend differentiation theory, found that internet use improved health outcomes for rural residents to a greater extent than for urban residents, demonstrating its compensatory value for populations traditionally disadvantaged in information access (21, 22). This finding is supported by research showing that internet access effectively narrows health disparities across regions and income groups by facilitating health information acquisition (23).

At the service compensation level, internet use enhances healthcare accessibility through telemedicine and online consultations (24–27). Studies have demonstrated that online health consultations overcome geographical barriers, enabling residents in underserved areas to access professional medical advice (28). Research has also found that the internet improves healthcare affordability for low-income groups (29), and that online appointment systems reduce healthcare costs for vulnerable populations (30, 31).

Concerning support compensation, internet use facilitates the formation of new social support networks that complement traditional support systems. Yang et al. showed that broadband access substantially increased social engagement among middle-aged and older adults, particularly providing crucial support for rural empty-nest seniors facing geographic isolation (32). Research has confirmed that digital literacy improves older adults’ health by strengthening social support, with more pronounced benefits among those with lower educational attainment (33, 34). Additional studies have documented that the internet enables rural older adults to access greater health support, thereby narrowing urban–rural resource gaps (35), and particularly enhances mental health among low-income older adults with chronic diseases (36). Evidence also indicates that internet use improves mental health among vulnerable groups by promoting social participation and enhancing remote family support (37, 38).

The contrasting findings from these two strands of literature indicate that the relationship between internet use and health inequality is context-dependent. The exacerbating effect tends to predominate when digital infrastructure remains unevenly distributed and when disadvantaged groups lack the skills to translate internet access into health benefits. The equalizing effect is more likely to emerge when internet penetration reaches sufficient levels and when compensatory mechanisms such as telemedicine and online social support are accessible to underserved populations.

Despite the growing body of evidence, most studies focus on urban–rural or generational comparisons, with limited attention to heterogeneity within specific populations such as farmers. Few studies have systematically examined the mechanisms through which internet use influences health inequality or the conditions under which different effects occur. These gaps are particularly significant in rural China, where rapid digital expansion presents both opportunities and risks for health equity.

This study makes four contributions to the literature. First, it extends the theoretical framework by moving beyond aggregate urban–rural comparisons to examine heterogeneity within the farmer population. Drawing on inclusive development theory, we assess whether internet use improves farmers’ overall health and whether these benefits are distributed equitably or concentrated among advantaged subgroups. Second, this study advances the methodology by utilizing six waves of panel data (2012–2022) from the China Family Panel Studies (CFPS). To address endogeneity, we employ an instrumental variable strategy using geographical characteristics of respondents’ villages as instruments, estimated via two-stage least squares (2SLS). This approach provides more robust causal identification than cross-sectional analyses prevalent in the existing literature. Third, this study deepens the understanding of mechanisms by examining two mediation pathways: the mediating role of healthcare accessibility and the mediating role of healthcare affordability in the relationship between internet use and health inequality among farmers. These models provide empirical tests of the compensatory mechanisms proposed in the literature. Fourth, this study enriches the heterogeneity analysis by examining how the effects of internet use vary across life cycle stages, educational attainment, and healthcare expenditure levels, offering actionable insights for targeted policy interventions.

## 3 Materials and methods

### 3.1 Study sample

#### 3.1.1 Data source and sample selection

The data used in this study are drawn from the China Family Panel Studies (CFPS), a large-scale, nationally representative longitudinal survey conducted by the Institute of Social Science Survey (ISSS) at Peking University. The CFPS employs a multi-stage stratified probability proportional to size (PPS) sampling design. At the first stage, administrative units (counties or districts) were selected as primary sampling units using PPS sampling, stratified by region across 25 provinces, municipalities, and autonomous regions, representing approximately 95% of China’s total population. At the second stage, villages or communities were selected within each primary unit. At the third stage, households were randomly selected, and all eligible members were interviewed face-to-face using computer-assisted personal interviewing (CAPI) technology (39).

This study utilizes data from six consecutive waves spanning a decade (2012–2022). Table 1 presents detailed information on each survey wave, including field periods and sample sizes. The 2010 baseline wave was excluded due to the absence of key variables related to internet usage. Notably, the 2020 survey employed telephone interviews as the primary follow-up method due to disruptions caused by the COVID-19 pandemic. To ensure the analytical focus on rural residents, we applied the following selection criteria: (1) excluding respondents with urban household registration (hukou); (2) excluding individuals under 18 years of age; (3) removing observations with missing values and outliers on key variables; and (4) excluding outliers identified through statistical diagnostics. After applying these criteria, the final analytical sample comprises 9,360 person-wave observations.

**Table 1:** Information on CFPS survey waves.

Wave	Survey year	Field period	Rural sample used
1	2012	July–November 2012	1,560
2	2014	July 2014–May 2015	1,560
3	2016	July 2016–May 2017	1,560
4	2018	June 2017–May 2018	1,560
5	2020	July–November 2020	1,560
6	2022	May–November 2022	1,560
		Total	9,360

#### 3.1.2 Sample characteristics

Table 2 presents the demographic and socioeconomic characteristics of the analytical sample. The sample is relatively balanced in terms of gender, with males accounting for 41.3% and females for 58.7%. The mean age of respondents is 50.6 years (SD = 12.8), with the largest proportion in the middle-aged group (45–59 years, 42.0%). In terms of educational attainment, the majority have relatively low levels of formal education, with 52.3% having completed 6 years or less of schooling. Regarding marital status, 89.2% of respondents are married. The geographic distribution is relatively balanced across Eastern (38.5%), Central (28.7%), and Western China (32.8%). Notably, 33.0% of rural respondents reported using the internet, while 95.0% reported having health insurance coverage.

**Table 2:** Descriptive statistics of sample characteristics (*N* = 9,360).

Variable	Category	N	%
Gender	Male	3,864	41.3
	Female	5,496	58.7
Age group	Young adults (18–44)	3,024	32.3
	Middle-aged adults (45–59)	3,927	42.0
	Older adults (60–89)	2,409	25.7
Education	≤6 years	4,892	52.3
	6–12 years	3,237	34.6
	>12 years	1,231	13.1
Marital status	Married	8,348	89.2
	Other	1,012	10.8
Region	Eastern	3,609	38.5
	Central	2,682	28.7
	Western	3,069	32.8
Internet usage	Yes	3,092	33.0
	No	6,268	67.0
Health insurance	Yes	8,891	95.0
	No	469	5.0

### 3.2 Measures

#### 3.2.1 Dependent variable

The dependent variable is health inequality among farmers, measured using the *Kakwani* relative deprivation index. This index was constructed based on respondents’ self-reported health status, which was assessed using the following question: “How would you rate your current health status?.” Responses were recorded on a five-point Likert scale: 1 = “Poor,” 2 = “Fair,” 3 = “Average,” 4 = “Good,” and 5 = “Excellent.” Higher scores indicate better self-reported health. The *Kakwani* relative deprivation index quantifies the aggregate gap between an individual’s health status and that of all individuals with better health within the reference group. For individual *i* with health status *y_i_*, the index (Equation 1) is calculated as follows:


$$\text{Kakwani}=\frac{1}{\mathrm{n\mu}}\sum_{j=i+1}^{n}(y_{1}-y_{i})$$ (1)

where *μ* denotes the average health level of the rural population, and *n* represents the total number of individuals in the sample. The index ranges from 0 to 1, with higher values indicating greater relative health deprivation (i.e., more disadvantaged position in the health distribution) (40, 41). For robustness checks, the *Yitzhaki* index (Equation 2) was used as an alternative measure:


$$\text{Yitzhaki}=\frac{1}{n}\sum_{j=i+1}^{n}(y_{1}-y_{i})$$ (2)

#### 3.2.2 Independent variable

The independent variable in this study is internet usage. This variable was measured based on respondents’ answers to the CFPS adult questionnaire item: “Do you use the internet (including mobile internet via smartphone)?.” Responses were coded as a binary variable: 1 = “Yes” and 0 = “No.” Since the 2012 wave did not include a direct question on internet usage, respondents’ internet use status for that year was inferred from related items regarding the frequency of computer use and mobile phone internet access.

#### 3.2.3 Mediating variables

Two mediating variables were examined to explore the mechanisms through which internet usage affects health inequality.

Accessibility of healthcare services. This variable was measured by respondents’ health insurance coverage status, based on the question: “Are you currently covered by any of the following health insurance programs: Public Medical Insurance (PMI), Urban Employee Basic Medical Insurance (UEBMI), Urban Resident Basic Medical Insurance (URBMI), Supplementary Medical Insurance (SMI), New Rural Cooperative Medical Scheme (NRCMS)?.” Responses were coded as 1 = “Yes” (covered by at least one type) and 0 = “No.”

Affordability of healthcare services. This variable was measured by the natural logarithm of per capita household income, calculated as total household income divided by household size. Total household income was assessed using the question: “What was your household’s total income last year, including wages, agricultural income, business income, transfers, and property income?.” The logarithmic transformation was applied to reduce skewness and facilitate interpretation.

#### 3.2.4 Control variables

Nine control variables were included at both individual and household levels. Individual-level controls include age, age squared (to capture nonlinear effects), gender, marital status, and years of education (42). Household-level controls include household size, per capita expenditure, housing assets, and land assets. Table 3 presents the definitions and measurements of all control variables.

**Table 3:** Definitions and measurements of control variables.

Variable	Survey question	Measurement
Age	What is your date of birth?	Continuous (years)
Age squared	Derived from Age	Age^2^ / 100
Gender	What is your gender?	1 = Male, 0 = Female
Marital status	What is your current marital status?	1 = Married, 0 = Other
Educational attainment	What is your highest educational attainment?	Illiterate/Semi-literate = 0; Elementary = 6; Junior High = 9; Senior High = 12; College = 15; Undergraduate = 16; Graduate = 19
Household size	Please indicate which of the following individuals are currently members of your household.	Total Number of Household Members (person)
Per capita household expenditure	What was your household’s total expenditure last year (covering all categories such as consumption, transfers, social insurance, and housing loans)?	Total Household Expenditure/ Household Size (yuan), take logarithm
Household housing assets	What is the total current market value of your primary residence and any other real estate properties owned by your household?	Total Household Housing Assets (yuan), take logarithm
Household land assets	What is the estimated total market value of your household’s land holdings?	Total Household Land Assets (yuan), take logarithm

### 3.3 Model settings

#### 3.3.1 Benchmark regression model

The impact of internet usage on farmers’ health may manifest in two ways. On one hand, internet use can directly promote improvements in farmers’ overall health through channels such as greater dissemination of health information and improved access to medical services. On the other hand, there are significant disparities among farmers in terms of internet access, digital skills, and the ability to convert resources into health benefits. As a result, the positive health effects of the internet may be distributed unevenly across different subgroups, which could potentially exacerbate health inequalities among farmers. Therefore, it is essential to first clarify the direction and magnitude of the impact of internet usage on the absolute health status of farmers in order to accurately assess the evolution of health inequality. This distinction enables us to determine whether internet use leads to widening or narrowing health disparities within a context of overall improvement, or whether it simply maintains pre-existing differences in the absence of such improvement. Accordingly, this paper first empirically examines the fundamental relationship between internet usage and farmers’ health levels, followed by a systematic exploration of the internet’s impact on health inequality among farmers. After conducting F-tests, LM tests, and Hausman tests on the model, this study selects a two-way fixed effects model—controlling for both time and region—as the benchmark regression model. The model specification is as follows (Equations 3, 4):


$$\text{Healt}h_{i,t}=\theta_{0}+\theta_{1}\text{Interne}t_{i,t}+\theta_{i}\text{Contro}l_{i,t}+\mu_{i}+\lambda_{t}+\varepsilon_{i,t}$$ (3)

$$\text{Kakwan}i_{i,t}=\beta_{0}+\beta_{1}\text{Interne}t_{i,t}+\beta_{i}\text{Contro}l_{i,t}+\mu_{i}+\lambda_{t}+\varepsilon_{i,t}$$ (4)

Where *i* and *t* denote province and year, respectively; $\text{Interne}t_{i,t}$ indicates farmers’ internet usage status; $\text{Healt}h_{i,t}$ and $\text{Kakwan}i_{i,t}$ denote farmers’ health status and health inequality, respectively; $\text{Contro}l_{i,t}$ represents a set of control variables; $\theta_{i}$ and $\beta_{i}$ are the parameters to be estimated; $\mu_{i}$ is the region fixed effect; $\lambda_{t}$ is the time fixed effect; and $\varepsilon_{i,t}$ is the random disturbance term.

#### 3.3.2 Mediating effect model

To examine the relationships among internet usage, healthcare accessibility, and health inequality—as well as among internet usage, healthcare affordability, and health inequality—among farmers, this study further tests whether internet usage alleviates health inequality by enhancing healthcare accessibility and affordability. The mediation effect testing method proposed by Wen and Ye is adopted for this purpose (43). Building on the baseline regression model, the following specification is constructed (Equations 5–8):


$$\text{Incom}e_{i,t}=\chi_{0}+\chi_{1}\text{Interne}t_{i,t}+\chi_{i}\text{Contro}l_{i,t}+\mu_{i}+\lambda_{t}+\varepsilon_{i,t}$$ (5)

$$\begin{array}{l} \text{Kakwan}i_{i,t}=\psi_{0}+\psi_{1}\text{Interne}t_{i,t}+\psi_{2}\text{Incom}e_{i,t}\\ +\psi_{i}\text{Contro}l_{i,t}+\mu_{i}+\lambda_{t}+\varepsilon_{i,t} \end{array}$$ (6)

$$\text{Trus}t_{i,t}=\partial_{0}+\partial_{1}\text{Interne}t_{i,t}+\partial_{i}\text{Contro}l_{i,t}+\mu_{i}+\lambda_{t}+\varepsilon_{i,t}$$ (7)

$$\begin{array}{l} \text{Kakwan}i_{i,t}=\kappa_{0}+\kappa_{1}\text{Interne}t_{i,t}+\kappa_{2}\text{Trus}t_{i,t}\\ +\kappa_{i}\text{Contro}l_{i,t}+\mu_{i}+\lambda_{t}+\varepsilon_{i,t} \end{array}$$ (8)

where $\chi_{i}$ , $\psi_{i}$ , $\partial_{i}$ , and $\kappa_{i}$ are the parameters to be estimated; $\text{Incom}e_{i,t}$ and $\text{Trus}t_{i,t}$ represent farmers’ income level and interpersonal trust, respectively; All other terms are as defined in Equation 3.

## 4 Results

### 4.1 Descriptive statistics and correlation analysis

Before presenting the main regression results, we first provide an overview of the key variables used in this study. Table 4 reports the descriptive statistics for all variables. The mean value of health inequality (*Kakwani* index) is 0.37, indicating that rural residents experience moderate levels of relative health deprivation on average. Regarding internet usage, 33.0% of respondents reported using the internet, reflecting the growing but still limited digital penetration in rural China during the study period. The average self-reported health score is 2.86, suggesting that most respondents perceive their health status as “relatively healthy.” Healthcare accessibility is relatively high, with 95.0% of respondents covered by at least one health insurance program. The mean age of respondents is 50.59 years, and the average years of education is 6.50, indicating that the sample primarily consists of middle-aged and older farmers with relatively low educational attainment.

**Table 4:** Descriptive statistics.

Variable type	Variable name	Standard error	Mean	Minimum	Maximum value
Dependent variable	Health Inequality Among Farmers (Kakwani Index)	0.32	0.37	0.00	1.00
	Health Inequality Among Farmers (Yitzhaki Index)	0.60	0.82	0.08	1.95
	Farmers’ health status	1.26	2.86	1.00	5.00
Core explanatory variables	Internet usage	0.47	0.33	0.00	1.00
Mediating variable	Access to healthcare services	0.22	0.95	0.00	1.00
	Affordability of Healthcare Services	1.06	9.16	5.66	11.52
Instrumental variables	Geographical and topographical characteristics	0.39	0.18	0.00	1.00
Control variables	Age	12.78	50.59	19.00	88.00
	Age squared	0.54	7.78	5.89	8.95
	Gender	0.49	0.59	0.00	1.00
	Marital Status	0.31	0.89	0.00	1.00
	Educational Attainment	4.21	6.50	0.00	19.00
	Household Size	1.34	3.04	1.00	12.00
	Per capita household expenditure	0.89	9.19	0.00	13.00
	Household Housing Assets	3.04	10.92	0.00	16.26
	Household Land Assets	4.25	7.88	0.00	14.80

Table 5 presents the Pearson correlation coefficients among the main variables. The results show that internet usage is positively correlated with farmers’ health status (*r* = 0.117, *p* < 0.01). Internet usage is also negatively correlated with health inequality (*r* = −0.048, *p* < 0.01), providing preliminary evidence that internet use may help reduce health disparities among farmers. Additionally, healthcare accessibility (*r* = −0.031, *p* < 0.01) and healthcare affordability (*r* = −0.045, *p* < 0.01) show significant negative correlations with health inequality, consistent with the expectation that healthcare accessibility and affordability contribute to reducing health disparities, while also providing a preliminary foundation for further exploration into how the internet influences health inequality through these pathways. The correlation analysis provides initial support for the hypothesized relationships but does not account for potential confounding factors. Therefore, we proceed with multivariate regression analysis to rigorously examine the causal effects of internet usage on health inequality among farmers.

**Table 5:** Correlation matrix of main variables.

Variable	(1)	(2)	(3)	(4)	(5)
(1) Health inequality among farmers	1.000				
(2) Farmers’ health status	0.022**	1.000			
(3) Internet usage	−0.048***	0.117***	1.000		
(4) Access to healthcare services	−0.031***	−0.029***	−0.003	1.000	
(5) Affordability of healthcare services	−0.045***	0.136***	0.382***	0.005	1.000

***p < 0.01, **p < 0.05, *p < 0.1.

### 4.2 Benchmark results

Based on the results of the Hausman test, this study adopts a two-way fixed effects model to empirically examine the impact of internet usage on farmers’ health levels and health inequality. The specific regression outcomes are presented in Table 6. Columns (1) and (2) report the effects of internet usage on farmers’ health levels after controlling for time and regional effects and progressively incorporating individual and household-level variables. The regression results indicate that the coefficient for internet usage is consistently positive and statistically significant at the 1% level of significance. This confirms that internet usage consistently improves farmers’ health status. This is consistent with the scholars’ conclusion that internet usage is beneficial to health (44–46). Columns (3) and (4) assess the impact of internet usage on health inequality among farmers. After controlling for time and regional effects, the models sequentially include individual and household-level control variables. The regression coefficients for the core explanatory variable (i.e., internet usage) are estimated at −0.038 and −0.037, with corresponding 95% confidence intervals of [−0.056, −0.020] and [−0.055, −0.018], respectively. Both are significantly negatively correlated at the 1% significance level. This suggests that internet usage has a strong mitigating effect on health inequality, meaning that greater internet penetration helps to reduce health disparities within the farming population. Overall, Table 6 demonstrates that internet usage not only directly enhances the overall health levels of farmers but also narrows health inequalities arising from information barriers and resource disparities. This illustrates a dual positive effect: promoting both health improvement and health equity. The underlying reason is that internet usage facilitates the sharing and dissemination of medical and health information, enabling farmers to more easily access health knowledge and medical resources. As a result, their health awareness and self-care capabilities are enhanced. At the same time, the internet improves access to healthcare services, thereby reducing health disparities caused by unequal access to information and resources.

**Table 6:** Baseline regression results.

Variable	(1)	(2)	(3)	(4)
	Farmers’ health status		Health inequality among farmers	
Internet usage	0.207^***^	0.204^***^	−0.038***	−0.037***
	(0.033)	(0.033)	(0.009)	(0.009)
Age	0.010	0.014**	−0.002	−0.004**
	(0.006)	(0.006)	(0.002)	(0.002)
Age quadratic term	−0.762^***^	−0.875***	0.049	0.081**
	(0.146)	(0.150)	(0.038)	(0.039)
Gender	0.188^***^	0.191^***^	−0.007	−0.005
	(0.027)	(0.027)	(0.007)	(0.007)
Marital status	0.013	−0.030	−0.025**	−0.018
	(0.041)	(0.044)	(0.011)	(0.011)
Educational attainment	0.035^***^	0.034^***^	−0.008***	−0.008***
	(0.003)	(0.003)	(0.001)	(0.001)
Household size		0.026^**^		−0.002
		(0.011)		(0.003)
Per capita household expenditure		−0.008		−0.005
		(0.017)		(0.004)
Household housing assets		0.010^**^		−0.003***
		(0.004)		(0.001)
Household land assets		−0.001		−0.002**
		(0.003)		(0.001)
Time fixed effect	Control	Control	Control	Control
Regional fixed effects	Control	Control	Control	Control
Constant term	2.447^***^	2.375^***^	0.210	0.127
	(0.045)	(0.163)	(0.217)	(0.223)
Observed value	9,360	9,360	9,360	9,360
*R* ^2^	0.048	0.049	0.022	0.023

***, **, and * denote significance levels of 1, 5, and 10%, respectively; standard errors are shown in parentheses.

The analysis of control variables is primarily based on the regression results from Model (4). The regression coefficient for age is significantly positive at the 1% level, indicating that health inequalities among farmers tend to widen as age increases. This may be due to declining physical function with age, which amplifies disparities in health resource demand and utilization. The coefficient for the age-squared term is also statistically significant at the 1% level but negative, indicating a nonlinear effect: the marginal impact of age on health inequality diminishes as age increases. The regression coefficient for gender is statistically significant and negative, suggesting that health inequalities exist between genders, with males potentially displaying different patterns of health inequality compared to females. The coefficient for marital status is also statistically significant and negative, implying that marital status influences health inequalities, with married individuals potentially exhibiting distinct patterns. Educational attainment shows a significantly negative coefficient at the 1% level, indicating that higher education helps to mitigate health inequalities among farmers. This may be because better-educated farmers possess greater health literacy, enabling them to manage their health more effectively and access health-related resources more easily. Among household-level variables, both housing assets and land assets have significantly negative coefficients, reflecting that household asset accumulation contributes to better health resource allocation and reduced health inequality. In contrast, household size and per capita household expenditure do not show significant effects, indicating that these factors did not significantly influence health inequality in this model.

### 4.3 Robustness checks

To ensure the reliability of the benchmark regression results, robustness tests were conducted from the following three perspectives:

1. Changing the Estimation Model: The dependent variable in this study, bounded within the interval [0, 1], exhibits a restricted nature with well-defined limits. The Tobit model is an appropriate estimation approach for such bounded dependent variables, as it efficiently incorporates both the continuous variation inside the interval and the mass points at the boundaries, leading to more accurate and efficient estimates. Accordingly, this study employs the Tobit model to re-estimate the empirical specifications. As shown in Column (1) of Table 7, the coefficient for internet usage is −0.037, which remains significantly negative at the 1% level. This result indicates that internet usage reduces health inequality among farmers, consistent with the findings of the benchmark regression.
2. Replacing the Dependent Variable: The Kakwani index is a derivative of the Yitzhaki index. In academic research, the application of the Yitzhaki index in lieu of the Kakwani index for robustness checks has become a widely accepted and prevalent practice. Therefore, this study substitutes the Kakwani index with the Yitzhaki index as the measure of health inequality. The regression results presented in Column (2) of Table 7 show no significant changes in either the direction or the significance of the coefficient for the core explanatory variable. This result further supports the conclusion that internet usage helps mitigate health inequality among farmers.
3. Excluding Municipal-Level Samples: Due to the distinct economic structures and policy environments of Beijing, Shanghai, Tianjin, and Chongqing, which may bias estimation results, this study re-estimates the model after excluding these municipalities. As reported in Column (3) of Table 7, the coefficient for internet usage remains significantly negative at −0.038. This result demonstrates that the research findings are robust and not driven by outlier samples.

**Table 7:** Robustness and endogeneity test results.

Variable	(1)	(2)	(3)	(4)
	Changing the estimation mode	Replacing the dependent variable	Excluding municipal-level samples	2SLS
Health inequality among farmers				
Internet usage	−0.037***	−0.059***	−0.038***	−0.041**
	(0.009)	(0.017)	(0.010)	(0.019)
Control variables	Control	Control	Control	Control
Regional solid effects	Control	Control	Control	Control
Time fixed effect	Control	Control	Control	Control
Constant term	0.076	−1.517***	0.062	0.078
	(0.235)	(0.408)	(0.226)	(0.235)
Observed value	9,360	9,360	9,100	9,360
*R* ^2^	0.050	0.059	0.024	0.014

***, **, and * denote significance levels of 1, 5, and 10%, respectively; standard errors are shown in parentheses.

### 4.4 Endogenous analysis

To address potential endogeneity issues arising from omitted variables or bidirectional causality, this study employs two-stage least squares (2SLS) estimation. Geographical terrain, which is exogenous to the economic system, is commonly used as an instrumental variable in empirical research. In this paper, the terrain type of the village or community where farmers reside is selected as the instrumental variable for internet usage. The assignment method is as follows: if the village or community is hilly, mountainous, a plateau, grassland, or a fishing village, the instrumental variable is coded as 0; if it is a plain rural area, it is coded as 1. From a causality perspective, complex topographies such as hills and mountains act as physical barriers, resulting in dispersed populations and higher infrastructure costs, as well as greater challenges for internet service coverage. This situation produces weaker network signals and lower service quality, directly limiting farmers’ internet access and usage—a condition that is reversed in rural areas. Therefore, there is a clear and robust causal relationship between topographical features and farmers’ internet usage, satisfying the causality condition for instrumental variables. Regarding exogeneity, topography is a naturally occurring characteristic with no direct connection to health inequalities among farmers. Furthermore, health inequalities cannot retroactively affect local geographic features, thus meeting the exogeneity requirement for instrumental variables. As shown in Column (4) of Table 7, after controlling for endogeneity, the coefficient for internet usage remains significantly negative. This finding further confirms the causal effect of internet usage in reducing health inequalities among farmers, consistent with earlier conclusions. In addition, the instrumental variable tests indicate that the Kleibergen-Paap rk LM test yields a *p*-value of 0, and the Kleibergen-Paap rk Wald F-statistic exceeds the 10% threshold for weak identification. These results indicate that there are no issues of under-identification or weak identification in the instrumental variables, providing further support for the study’s hypothesis.

### 4.5 Analysis of mediation effect

To investigate whether internet usage alleviates health inequalities among farmers by improving the accessibility and affordability of healthcare services, this study employs a mediation model to test the relationships among internet usage, healthcare accessibility, and health inequalities, as well as among internet usage, healthcare affordability, and health inequalities. Table 8, Column (1), presents the results for the impact of internet usage on farmers’ healthcare accessibility. The regression coefficient for the core explanatory variable—internet usage—is 0.013 and is significant at the 5% level, indicating that internet usage has a significant impact on enhancing healthcare accessibility for farmers. Column (2) reports the total effect of internet usage on health inequality among farmers after incorporating the mediating variable of healthcare accessibility. The coefficient for internet usage is −0.036, which is significantly negative at the 1% level, while the coefficient for healthcare accessibility is −0.032, also significantly negative at the 5% level. The empirical results from Columns (1) and (2) indicate that internet usage reduces health inequalities among farmers by improving access to healthcare services. The underlying mechanisms can be summarized in three main aspects. First, internet usage effectively breaks down information barriers, enabling the efficient dissemination of health education, disease prevention knowledge, medical insurance reimbursement procedures, and information on designated medical institutions throughout rural areas. This reduces information asymmetry and helps ensure that farmers do not miss critical treatment opportunities. Second, internet usage expands access to healthcare channels; features such as remote consultations, online appointment booking, and medication delivery significantly reduce transportation and time costs for farmers in remote areas. As a result, they can access higher-quality medical services without being limited by local primary healthcare resources. Third, internet usage enhances equity in accessing healthcare resources. Even low-income or less-educated farmers can easily utilize these services via simplified interfaces, helping to overcome exclusion from formal healthcare due to barriers such as complex offline procedures or communication difficulties. By bridging gaps in healthcare accessibility among different farmer groups, internet usage mitigates health disparities arising from difficulties in accessing medical care when needed and thus effectively alleviates health inequalities within the farming population.

**Table 8:** Results of mediation effect test.

Variable	(1)	(2)	(3)	(4)
	Access to healthcare services	Health inequality among farmers	Affordability of healthcare services	Health inequality among farmers
Internet usage	0.013**	−0.036***	0.184***	−0.035***
	(0.006)	(0.009)	(0.025)	(0.010)
Access to healthcare services		−0.032**		
		(0.015)		
Affordability of healthcare services				−0.011***
				(0.004)
Control variables	Control	Control	Control	Control
Regional solid effects	Control	Control	Control	Control
Time fixed effect	Control	Control	Control	Control
Constant term	0.170	0.132	1.465**	0.143
	(0.152)	(0.222)	(0.591)	(0.223)
Observed value	9,360	9,360	9,360	9,360
*R* ^2^	0.021	0.024	0.368	0.024

***, **, and * denote significance levels of 1, 5, and 10%, respectively; standard errors are shown in parentheses.

Column (3) of Table 8 presents the estimated effects of internet usage on the affordability of medical services for farmers. The coefficient for the core explanatory variable—internet usage—is 0.184, which is significantly positive at the 1% level, indicating that internet usage substantially enhances the affordability of medical services for farmers. Column (4) further examines the total effect of internet usage on health inequality among farmers after introducing the mediating variable of medical service affordability. Here, the coefficient for internet usage is −0.035, which is significantly negative at the 1% level, while the coefficient for interpersonal trust is −0.011, which is also significantly negative at the 1% level. These results suggest that internet usage can help reduce health inequality among farmers by improving the affordability of medical services. The underlying mechanisms can be summarized as follows. First, internet usage effectively broadens income-generating channels for farmers. By utilizing e-commerce platforms to overcome geographical constraints in agricultural product sales, farmers can increase their operational income through better prices and higher sales volumes. Additionally, online labor platforms connect farmers with well-paying, flexible job opportunities, thereby further boosting their labor income. This income growth directly enhances farmers’ ability to afford healthcare, making medical expenditures a more manageable proportion of their income. As a result, farmers are better able to cover treatment costs when ill, preventing care delays due to insufficient income and reducing health disparities associated with deteriorating conditions. Second, internet usage improves farmers’ production and operational efficiency. Through the application of agricultural IoT technologies and online technical training, farming and breeding techniques are optimized, resulting in increased agricultural output and quality, while also reducing production costs. This income growth significantly improves the affordability of medical care within the constraints of farmers’ incomes. Even when medical expenditures arise, income support allows farmers to access necessary healthcare promptly, mitigating unequal access to care due to differences in payment capacity. Third, internet usage helps farmers manage income risks. By leveraging online services such as agricultural weather alerts and market trend forecasts, farmers can proactively respond to natural disasters and market fluctuations, stabilizing their income sources. Concurrently, online financial services offer credit support to address production funding gaps, ensuring a continuous income stream. This stable and growing income base serves as a solid foundation for healthcare expenditures, preventing farmers from falling into medical crises due to income interruptions or insufficiency. Consequently, it narrows the health inequality gap that arises from disparities in affordability.

### 4.6 Analysis of heterogeneity

#### 4.6.1 Heterogeneity across the life course

The impact of internet usage on health inequalities among farmers varies by age group. Differences in lifestyle, health needs, and levels of internet acceptance and utilization among farmers of different ages lead to distinct effects of internet usage on health inequalities. To investigate this heterogeneity, this section categorizes farmers by age according to the new segmentation proposed by the World Health Organization (WHO): young adults (44 years and younger), middle-aged adults (45–59 years), young older adults (60–74 years), and older adults (75–89 years). This classification is used to examine how internet usage affects health inequalities among farmers across these different age groups.

As shown in the regression results for columns (1), (2), and (3) of Table 9, the regression coefficient for the impact of internet usage on health inequalities among young farmers is −0.056, which is significantly negative at the 1% level. For middle-aged individuals, the coefficient is −0.045, which is also significantly negative at the 1% level. In contrast, for older adults, the effect is not significant. These findings indicate that the mitigating effect of internet usage on health inequalities among farmers is strongest for young adults, followed by middle-aged adults, with no significant effect observed among older adults. This pattern can be explained by differences in internet acceptance and proficiency across age groups. Young adults tend to have higher acceptance and skill in using the internet, enabling them to fully leverage it to access health information and medical services. This not only improves their own health status but also helps narrow the health gap between groups. Middle-aged individuals possess a moderate level of internet proficiency, allowing them to benefit from online resources, although their utilization is somewhat less extensive than that of young adults. In contrast, older adults, due to limited familiarity and operational proficiency with the internet, are less able to benefit from it for health improvement, resulting in a non-significant impact on their health inequalities.

**Table 9:** Results of heterogeneity analysis.

Variable	(1)	(2)	(3)	(4)	(5)	(6)	(7)	(8)	(9)
	Young adults (18–44)	Middle-aged adults (45–59)	Older adults (60–89)	6 years or less	6 to 12 years	12 years and above	Low per capita household healthcare expenditure	Medium per capita household healthcare expenditure	Higher per capita household healthcare expenditure
Health inequality among farmers									
Internet usage	−0.056***	−0.045***	0.022	−0.027*	−0.049***	0.011	−0.056***	−0.030*	−0.032**
	(0.018)	(0.014)	(0.022)	(0.014)	(0.016)	(0.028)	(0.018)	(0.016)	(0.015)
Control Variables	Control	Control	Control	Control	Control	Control	Control	Control	Control
Regional Solid Effects	Control	Control	Control	Control	Control	Control	Control	Control	Control
Time Fixed Effects	Control	Control	Control	Control	Control	Control	Control	Control	Control
Constant term	0.581***	0.572***	0.490***	−0.412	0.271	1.287**	0.441	−0.173	−0.313
	(0.089)	(0.066)	(0.073)	(0.370)	(0.414)	(0.560)	(0.406)	(0.395)	(0.351)
Observed Value	3,024	3,927	2,409	4,892	3,237	1,231	3,120	3,120	3,120
*R* ^2^	0.023	0.018	0.035	0.015	0.013	0.029	0.025	0.021	0.031

***, **, and * denote significance levels of 1, 5, and 10%, respectively; standard errors are shown in parentheses.

#### 4.6.2 Heterogeneity in educational attainment

The impact of internet usage on health inequalities among farmers differs according to educational attainment. Farmers with varying levels of education display differences in internet comprehension, usage skills, and health awareness, resulting in distinct effects of internet usage on health inequalities. To investigate this heterogeneity, this section categorizes farmers into three educational groups: 6 years or less, 6 to 12 years, and 12 years or more. It then examines how the impact of internet usage on health inequalities manifests across these educational categories.

As shown in the regression results in columns (4), (5), and (6) of Table 9, the impact of internet usage on health inequalities among farmers varies by educational attainment. For the group with 6 years or less of education, the regression coefficient is −0.027, which is significantly negative at the 10% level of significance. For the 6- to 12-year-old group, the coefficient is −0.049, which is significantly negative at the 1% level, whereas for the group with 12 years or more, the effect is not significant. These findings indicate that the effect of internet usage in reducing health inequalities is strongest among those with 6 to 12 years of education, followed by those with 6 years or less of education, with no significant effect observed for those with 12 years or more of education. The underlying reasons are as follows. For groups with lower educational attainment (6 years or less), internet usage provides a significant compensatory effect. This group faces considerable disadvantages in health literacy and access to resources. The low barriers to entry of internet use help them overcome knowledge gaps, greatly improving access to health resources and thus effectively reducing health inequalities. For the group with medium education levels (6 to 12 years), internet usage leads to efficient conversion and amplification effects. These farmers not only receive information but also possess the skills to screen, understand, and apply digital health resources. This enables them to translate technological advantages into substantial health benefits, resulting in a “1 + 1 > 2” amplification effect. Their role in bridging health gaps is even more pronounced than that of the low-education group. For the group with higher education levels (12 or more years), internet usage brings only marginal improvements and is constrained by the “health ceiling effect.” This group already has high health literacy and diverse resource channels, so internet use merely supplements existing resources without driving significant change. To further improve health equity for this group, comprehensive structural reforms are necessary, extending beyond technological solutions.

#### 4.6.3 Heterogeneity in healthcare expenditures

The impact of internet usage on health inequalities among farmers is shaped by differences in per capita household healthcare expenditure. Households with varying levels of healthcare spending differ in their capacity for health investment, access to healthcare services, and utilization rates, resulting in distinct effects of internet usage on health inequalities. To investigate this heterogeneity, this section divides the sample into three equal groups according to household per capita healthcare expenditure: low, medium, and high. This approach enables an examination of how internet usage affects health inequalities among farmers with different levels of healthcare spending.

As shown in the regression results for columns (7), (8), and (9) of Table 9, the coefficient for the impact of internet usage on health inequalities among farmers in the low-expenditure group is −0.056, which is significantly negative at the 1% level of significance. For the medium-expenditure group, the coefficient is −0.030, which is significantly negative at the 10% level, while for the high-expenditure group, the coefficient is −0.032, which is significantly negative at the 5% level. These results indicate that the mitigating effect of internet usage on health inequalities is strongest among those with lower per capita household healthcare expenditure, followed by those with higher expenditure, and is least pronounced among those with medium expenditure. The underlying reasons are as follows. Farmers with lower per capita healthcare expenditure have limited capacity for health investment and often face greater challenges in accessing traditional health services. Internet usage—such as online consultations and affordable health education—offers more convenient and economical ways to access health services, significantly improving the accessibility of health resources and thus effectively reducing health inequalities. For farmers with higher per capita healthcare expenditure, while they possess some capacity for health investment, internet usage can further alleviate health inequalities by increasing the efficiency of service access and providing diverse health management tools. In contrast, farmers with medium per capita healthcare expenditure may be in an intermediate position, finding themselves neither highly constrained nor fully advantaged in accessing both traditional and internet-based health services. As a result, the effect of internet usage in this group is relatively weaker compared to the low- and high-expenditure groups.

## 5 Discussion

### 5.1 Key findings

This paper investigates the impact of internet usage on health outcomes and health inequality among farmers. First, internet usage demonstrates a significant positive effect on farmers’ health status, with a regression coefficient of 0.204 that is statistically significant at the 1% level. Concurrently, it significantly reduces health inequality among farmers, as indicated by a coefficient of −0.037, also significant at the 1% level. These core findings are consistent with existing literature. For example, empirical studies by Tian, utilizing urban–rural data from China, and Yu and Meng, based on cross-national data, have both confirmed that internet access can improve population health by reducing barriers to health information and disseminating scientific health knowledge, while also exerting an equalizing effect among disadvantaged groups (22, 23). By focusing specifically on the farming population, this study further validates the generalizability of this effect in rural contexts, providing a quantitative basis for the coordinated implementation of “Healthy Village” policies.

Second, this research identifies two mediating pathways through which the internet influences health inequality among farmers: (1) by enhancing healthcare service accessibility (mediation effect coefficient = −0.032, significant at the 5% level); and (2) by improving healthcare service affordability (mediation effect coefficient = −0.011, significant at the 1% level). This mechanism analysis provides a nuanced extension to prior research. The existing literature has primarily emphasized the mediating role of healthcare accessibility, as seen in studies such as Yoo, Zhong et al., and Liao and Luo (28–30). Beyond this, our study incorporates the critical dimension of healthcare affordability. This finding closely aligns with the dual challenges of “difficulty in accessing healthcare” and “high cost of healthcare” faced by farmers, providing policymakers with a clear and specific direction for accurately leveraging digital tools to solve the aforementioned problems. The results demonstrate that internet usage can enhance healthcare accessibility through functions such as online consultations and appointment booking, while also effectively expanding farmers’ income-generating opportunities. Stable income, in turn, provides a safeguard for medical expenditures, thereby increasing the affordability of healthcare services.

Finally, the study reveals heterogeneous effects of internet usage on health across different subgroups. From an age perspective, the health equalizing effect is most pronounced among young farmers (coefficient = −0.056), followed by middle-aged farmers (coefficient = −0.045); the effect among older farmers is not statistically significant. This finding contrasts with the conclusions of scholars such as Yang et al. and Yuan, who suggested that the health-enhancing effect of internet usage is stronger for the older adults (32, 36). This suggests that some older farmers may not be able to effectively leverage the internet to obtain health benefits or to narrow the health gap with other age groups, due to factors such as limited digital literacy and underdeveloped usage habits. In terms of educational attainment, the effect is strongest among farmers with 6–12 years of education (coefficient = −0.049), which is consistent with the finding of Chen and Wang (2025) that “the health-promoting effect of internet usage is more pronounced among low-education groups” (34). Regarding healthcare expenditure, the mitigating effect on health inequality is most notable in low-expenditure groups (coefficient = −0.056). This finding highlights the “shortfall-reducing” role of internet usage from the perspective of medical expenditure, thereby addressing a gap in the existing literature.

### 5.2 Add findings

The results from all three methods demonstrate that internet usage significantly reduces health inequality among farmers, with coefficients of −0.037, −0.059, and −0.038, all statistically significant at the 1% level. These findings confirm the robustness of the study’s conclusions. Furthermore, to address potential reverse causality between internet usage and farmers’ health status, previous studies have predominantly employed internet penetration rates as instrumental variables (12). This study introduces an innovative approach by using the topographic type of respondents’ villages as an instrumental variable, based on the rationale that topography is innate and stable, entirely independent of individual health behaviors and status, and not subject to reverse causality. This method more effectively addresses endogeneity concerns, thereby enhancing the credibility of causal inference. Estimation results from the two-stage least squares (2SLS) method indicate that internet usage has a significant effect on health inequality among farmers, with a coefficient of −0.041.

In addition, the policy implications derived from the heterogeneity analysis represent a noteworthy contribution. While existing research often concludes with descriptive analyses of heterogeneous characteristics, this study identifies specific intervention priorities for different groups based on heterogeneity results. For example, for young and middle-aged farmers with basic digital literacy, emphasis should be placed on promoting smart health management tools and online fitness guidance programs to foster proactive health management behaviors. For older farmers facing barriers to digital technology adoption, a comprehensive support model that combines “online age-friendly adaptations” with “offline assistance” should be established. For highly educated farmers, specialized services such as expert online consultations and personalized health plan customization should be offered. For groups with low healthcare expenditure, policies should focus on expanding access to preventive healthcare services. These findings provide precise empirical evidence to support the formulation of differentiated digital health intervention policies for rural areas, effectively addressing the limitation in existing research that emphasizes conclusions over application.

### 5.3 Strengths and limitations

This study has several notable limitations. First, to ensure sample continuity and comparability across years, the data range was limited to the period from 2012 to 2022. While this approach helps control for temporal variation, it may have resulted in the exclusion of some sample information from the 2010 baseline survey, potentially affecting sample integrity. Second, due to limitations in questionnaire design, the core explanatory variable—“internet usage”—was chosen for its operational simplicity. However, this measure does not distinguish between the frequency, type, or quality of internet use. Third, because of data constraints, the measurements of healthcare accessibility and affordability in this study could not incorporate broader societal indicators, thereby limiting the scope for more robust and nuanced analyses. Future research should utilize more granular survey data to refine the measurement of internet usage, including dimensions such as frequency, content type, and proficiency. This would enable a more in-depth examination of the health effects resulting from usage heterogeneity. Additionally, incorporating societal-level healthcare indicators would help establish a more comprehensive measurement system, providing stronger empirical support for research conclusions.

## 6 Conclusion

This paper draws on data from six waves of the China Family Panel Studies (CFPS) conducted over a decade (2012–2022) to construct an index of health inequality among farmers. It systematically investigates the impact of internet usage on health inequality and explores the underlying mechanisms. The main conclusions are as follows. First, baseline regression results show that the estimated coefficients for the impact of internet usage on farmers’ health status and on health inequality among farmers are 0.204 and −0.037, respectively, both statistically significant at the 1% level. This indicates that internet usage significantly improves health outcomes for rural residents, while concurrently reducing health inequalities within this population. This conclusion remains robust after accounting for endogeneity and conducting a series of robustness checks. Second, mediation analysis demonstrates that internet usage narrows health disparities not only by enhancing access to healthcare services (mediation effect coefficient = −0.032, significant at the 5% level) but also by improving their affordability (mediation effect coefficient = −0.011, significant at the 1% level). Third, heterogeneity analysis reveals marked group differences in the effect of internet usage on health inequalities among farmers. Across age groups, the mitigating effect is strongest for young farmers (coefficient = −0.056), then middle-aged ones (coefficient = −0.045), but statistically insignificant for older adults. Among farmers by education, the effect is most pronounced among farmers with 6 to 12 years of schooling (coefficient = −0.049). By healthcare expenditure, the impact is greatest for low-expenditure farmers (coefficient = −0.056), followed by high-expenditure groups, with a relatively limited effect among medium-expenditure groups.

Based on these findings, the following policy recommendations are offered:

First, strengthen the development and dissemination of rural digital infrastructure to address the persistent digital divide. Empirical evidence from this study demonstrates that internet usage significantly reduces health inequality among farmers, underscoring the importance of expanding digital access in rural areas. However, according to the China Internet Network Information Center (CNNIC), even though rural internet penetration reached 67.4% by 2024, substantial disparities remain between eastern and western regions, as well as between younger and older generations. Instrumental variable analysis further confirms that geographical terrain significantly constrains internet access in mountainous and remote areas. To address these challenges, policymakers should continuously upgrade rural network infrastructure, prioritizing coverage expansion in remote villages and economically disadvantaged areas. Broadband speeds and mobile communication signal quality should be improved to eliminate both geographical and service-quality barriers to internet access. Additionally, digital inclusion initiatives should be implemented to reduce rural internet fees through government subsidies and public-private partnerships, drawing on experiences from the European Union’s Digital Decade strategy and the United States’ Rural Digital Opportunity Fund. Regular digital skills training sessions should be conducted at village service centers, emphasizing practical applications such as searching for health information and utilizing online healthcare services. These measures will substantially enhance farmers’ internet access and digital literacy, establishing a robust foundation for digital health empowerment.

Second, leverage the internet to enhance both the accessibility and affordability of healthcare services. Mediation analysis from this study reveals that internet usage reduces health inequality through two primary pathways: improving healthcare accessibility and increasing healthcare affordability. To maximize these mediating effects, it is essential to further develop and promote Internet Plus Healthcare initiatives. Local governments should establish telemedicine platforms within county-level medical alliances to facilitate the delivery of high-quality medical resources to grassroots areas. Additional improvements to rural e-commerce pharmaceutical delivery systems and online medical consultation networks will allow farmers to conveniently access professional medical resources. This strategy aligns with the successful expansion of telehealth in the United States during the COVID-19 pandemic, which significantly improved healthcare access in rural populations. To enhance affordability, policy measures should capitalize on the income-generating potential of the internet by promoting rural e-commerce, advancing digital inclusive finance, and expanding online vocational training to increase farmers’ income. Furthermore, eligible internet-based medical services should be included in medical insurance coverage, and internet platforms should be used to increase awareness of insurance policies and critical illness assistance programs, thereby alleviating the financial burden of medical expenditures for farmers.

Third, implement targeted digital health intervention strategies tailored to specific population groups. Heterogeneity analysis from this study reveals substantial group-specific differences in the effect of internet usage on health inequality. The mitigating effect is most pronounced among young farmers, those with primary to secondary education, and those with low healthcare expenditures, while it is limited or insignificant among older farmers, those with higher education, and medium-expenditure groups. These findings underscore the need for differentiated, rather than uniform, policy interventions. For digitally proficient young and middle-aged adults, emphasis should be placed on promoting smart health management tools and online fitness programs to foster proactive health management behaviors. For older populations who face greater challenges in digital adoption, an integrated model that combines online age-friendly adaptations with offline assistance should be adopted, including simplified digital interfaces and personalized guidance from village doctors and volunteers—an approach consistent with the EU’s Active and Assisted Living Program. Regarding educational attainment, streamlined and scenario-based digital products, such as short videos and voice consultations, should be developed for groups with lower education levels, while specialized services, including expert consultations and personalized health plans, should be offered to highly educated individuals. For low-expenditure groups, policies should prioritize preventive healthcare by disseminating free educational content and promoting affordable health monitoring devices, ensuring that digital health acts as an equalizer rather than an amplifier of existing disparities.

Fourth, address online health risks and strengthen digital health literacy. While this study demonstrates the positive effects of internet usage on health outcomes and health equity, policymakers must also acknowledge the potential risks associated with digital health information. The proliferation of health misinformation online represents a significant threat, particularly for farmers with limited digital literacy who may be susceptible to false claims regarding disease prevention and unproven treatments. Additionally, the rapid advancement of artificial intelligence in healthcare introduces concerns about data privacy, algorithmic bias, and AI-generated misinformation. To mitigate these risks, comprehensive digital health literacy programs should be implemented to equip farmers with the skills necessary to identify credible health sources and recognize misinformation. Given the significant variation in the effects of internet usage by age and education, these programs should be tailored accordingly, with simplified, visually based training for older and less-educated populations, and more advanced critical evaluation skills for younger groups. Regulatory frameworks should also be established to ensure the quality and safety of online health information and AI-powered health applications, drawing on the EU’s Digital Services Act and AI Act as references. Only by balancing the promotion of internet usage with appropriate risk management can digital technologies truly contribute to health equity among farmers.

## Data Availability

The original contributions presented in the study are included in the article/supplementary material, further inquiries can be directed to the corresponding author.
